# Changes in the Distribution of Pectin in Root Border Cells Under Aluminum Stress

**DOI:** 10.3389/fpls.2019.01216

**Published:** 2019-10-02

**Authors:** Teruki Nagayama, Atsuko Nakamura, Naoki Yamaji, Shinobu Satoh, Jun Furukawa, Hiroaki Iwai

**Affiliations:** ^1^Graduate School of Life and Environmental Sciences, University of Tsukuba, Tsukuba, Japan; ^2^Faculty of Life and Environmental Sciences, University of Tsukuba, Tsukuba, Japan; ^3^Research Institute for Bioresources, Okayama University, Chuo, Kurashiki, Japan

**Keywords:** aluminium, root border cells, pectin, mucilage, rice (*Oryza sativa* L.)

## Abstract

Root border cells (RBCs) surround the root apices in most plant species and are involved in the production of root exudates. We tested the relationship between pectin content in root tips and aluminum (Al) tolerance by comparing these parameters in wild-type (WT) and sensitive-to-Al-rhizotoxicity (*star1*) mutant rice plants. Staining for demethylesterified pectin decreased after Al treatment in the WT. A high level of pectin was observed in RBCs of the root tips. The level of total pectin was increased by about 50% compared with the control. In the Al-sensitive *star1* mutant, Al treatment decreased root elongation and pectin content, especially in RBCs. In addition, almost no Al accumulation was observed in the control, whereas more Al was accumulated in the RBCs of STAR1 roots. These results show that the amount of pectin influences Al tolerance; that Al accumulation in rice roots is reduced by the distribution of pectin in root-tip RBCs; and that these reactions occur in the field around the RBCs, including the surrounding mucilage. Al accumulation in rice roots is reduced by the distribution of pectin in root tips, and pectin in the root cell walls contributes to the acquisition of Al tolerance in rice by regulating its distribution. The release of Al-binding mucilage by RBCs could play a role in protecting root tips from Al-induced cellular damage.

## Introduction

Aluminum (Al) is the most abundant metal in the Earth’s crust. Under acidic conditions, Al is solubilized to its ionic form, which is toxic to plants (Foy, 1988). Al rapidly inhibits root elongation and, subsequently, the uptake of water and nutrients, resulting in significant reductions in crop production in acidic soils, which comprise 30% to 40% of the world’s arable soils (Von Uexküll and Mutert, 1995). Al compounds are affected by the pH of coexisting elements and soil. Under normal environmental conditions, Al assumes the form of Al(OH)_4_^−^; with decreasing pH, its morphology changes to Al(OH)_3_, Al(OH)_2_, and Al(OH)^2+^ (Duan and Gregory, 2003). In an acidic soil with pH <5, it elutes in the form of the water-soluble ion Al^3 +^, which is believed to most strongly inhibit the growth of plants when absorbed (Famoso et al., 2010). Growth inhibition by Al has been reported in various species, including rice, wheat, corn, rye, beet, mushroom, tomato, and *Arabidopsis thaliana* (Searcy and Mulcahy, 1990; Li et al., 2000; Famoso et al., 2010; Parra-Almuna et al., 2018; Xu et al., 2018). The toxicity mechanisms of Al are complex, and the exact mechanism by which Al initially causes the inhibition of root elongation is not fully understood (Kochian et al., 2005). However, most Al-related events clearly result from the binding of Al to extracellular and intracellular substances because of the high affinity of Al for oxygen donor compounds. Most Al inhibiting root elongation are localized to cell walls in the epidermis and outer cortex (Jones et al., 2006).

Some studies of the localization of Al in the cell wall have involved the examination of its relationship to pectin (Chang et al., 1999) and hemicellulose (Yang et al., 2011; Zhu et al., 2017a; Zhu et al., 2017b; Xu et al., 2018) contents. Meanwhile, some plant species have developed mechanisms to cope with internal and external Al toxicity (Ma et al., 2001; Ryan et al., 2001; Rengel, 2004; Kochian et al., 2005). Some plants are Al tolerant, and several Al resistance mechanisms have been proposed in previous studies. For example, buckwheat has been shown to transport absorbed Al to the aerial part of the plant, which then accumulates in vacuoles in a state chelated to oxalic acid (Wang et al., 2015). The most documented mechanism of Al resistance is the secretion of organic acid anions from plant roots (Li et al., 2000; Ma, 2000; Ma et al., 2001; Ryan et al., 2001; Kochian et al., 2005; Hoekenga et al., 2006; Kopittke et al., 2017). Root border cells (RBCs) and mucilage have also been suggested to increase Al tolerance in plants (Cai et al., 2011; Yang et al., 2016). RBCs are living cells that detach from the root cap and are considered to be involved in stress responses to the soil and produce hydrophilic polysaccharides in the soil. Polysaccharides secreted in the soil are hydrated and become mucilage substances (Hawes and Lin, 1990; Yu et al., 2009). RBCs are suggested to have different gene expression and metabolism pattern from cells in root tips (Watson et al., 2015). RBCs and mucilages have been shown to protect root tips from chemical and biological stresses, such as iron toxicity (Xing et al., 2008) and fungal infection (Hawes et al., 2000; Gunawardena and Hawes, 2002). Previous reports showed that the presence of RBCs and mucilages decreases Al accumulation and increases Al tolerance (Barcelo and Poschenrieder, 2002; Curlango-Rivera et al., 2016; Hawes et al., 2016). Al-resistant plants tend to have less Al accumulation than do Al-sensitive plants (Ma et al., 2005). In addition, the cell wall has been shown to readily adsorb and bind to Al (Van et al., 1994). Rice is one of the world’s major crops, and it has shown relatively high Al tolerance (Famoso et al., 2010). However, the mechanism responsible for Al tolerance in rice is not well understood. Rye has been suggested to have Al resistance equivalent to that of rice, with organic acid secretion from the root ends (Li et al., 2000). Increased Al concentrations also increase citric acid secretion in rice, with no significant difference in this secretion between Al-resistant varieties (Koshihikari) and Al-sensitive varieties (Katalath) (Ma et al., 2002). Moreover, no significant decrease in Al tolerance has been observed in mutants with little organic acid secretion (Yokosho et al., 2016). These findings suggest that the secretion of organic acids is not strongly involved in the Al resistance of rice. In recent years, gene expression studies have been undertaken to investigate the mechanism of Al tolerance in rice, and the transcription factor ADP-ribosyltransferase 1 (ART1), whose expression is increased by Al stress, has been identified. Furthermore, the sensitive to Al rhizotoxicity 1 (STAR1) gene encoding the ABC transporter has been shown to be among several genes whose expression is controlled by ART1 (Huang et al., 2009). These genes are thought to contribute to rice Al resistance (Delhaize et al., 2012), as ART1 and the *star1* mutants are strongly susceptible to growth inhibition by Al absorption (Delhaize et al., 2012). The *star1* mutant has a DNA region that is thought to encode the nucleotide-binding domain. It is expressed throughout the roots, excluding the mature epidermis, suggesting localization to vesicles in cells. STAR1 has been suggested to form a complex with STAR2, which has a transmembrane domain and specifically transports UDP glucose. UDP glucose transported outside of cells from vesicles is used for the modification of cell wall polysaccharides, and Al stress is thought to be reduced by the modification of binding between Al and these polysaccharides (Huang et al., 2009). In a study comparing changes in gene expression in response to Al treatment (Tsutsui et al., 2012), elevated expression of galacturonosyltransferase, a gene involved in the biosynthesis of pectin 1 to 2 cm from the root tips, was observed in wild-type (WT) but not in STAR1 plants.

In this study, we examined the relationship between pectin distribution in root tips and plant Al tolerance using hydroponics. The Al concentration we used was higher than the acid soil, but consistent with previous Al toxicity researches in rice. Our results show that the pectin distribution influences Al tolerance, that Al accumulation in rice roots is reduced by the distribution of pectin in root tips, and that pectin in the root cell walls contributes to the acquisition of Al tolerance in rice by regulating its distribution.

## Materials and Methods

### Plant and Growth Conditions

Nipponbare, Koshihikari, and *star1* mutant of rice (*Oryza sativa*) were used in this study (Huang et al., 2009). After imbibition in ion exchanged water for 1 or 3 days at 30°C, rice seedlings were grown on floating net on 1.0 or 0.5 mM CaCl_2_, pH 4.5 for 3 days. Grown seedlings were exposed to Al with 1.0 or 0.5 mM CaCl_2_ and 0, 50, or 100 µM AlCl_3_, pH 4.5 water culture media for 1 day in 15-ml centrifuge tube. Free Al activities were evaluated by using GEOCHEM-EZ software (Yoshida et al., 1972; Shaff et al., 2010) and values are between 76.57% and 78.28%. They were grown at 30°C under continuous light at 250 μmol m^−2^ s^−1^.

### Measurement of Root Elongation

Root length was measured before and after Al treatment with a ruler to calculate root elongation during Al treatment. Relative root elongation, RRE (%) = (root growth in each Al condition)/(root growth in control) × 100 was calculated to compare root elongation and Al tolerance between different lines (Furukawa et al., 2007).

### Saponification of Pectin

To remove the methyl groups from pectin and change methylesterified pectin to demethylesterified pectin, methylesterified pectin in roots was saponified with 0.1 N NaOH in 50-ml centrifuge tube for 1 min. After saponification, roots were washed with ion exchanged water, and then all pectin can stain by ruthenium red (Iwai et al., 1999).

### Staining Demethylated Pectin With Ruthenium Red

To detect the demethylesterified pectin in roots, sample roots were stained with 0.01% (w/w) ruthenium red in 50-ml centrifuge tube for 5 min. After staining, roots were washed with ion exchanged water (Iwai et al., 1999).

### Staining Al With Eriochrome Cyanine R

Al in roots was stained with 0.1% (w/w) eriochrome cyanine R in 50-ml centrifuge tube for 20 min. After staining, roots were washed with ion exchanged water (Jones and Thurman, 1957).

### Collecting Cell Wall

Root tips (0–1 mm) from three seedlings were cut with a razor and collected in a 2.0-ml tube as a cell wall sample. Samples were frozen in liquid nitrogen and crushed with pestles.

A series of processes was repeated twice, adding methanol/chloroform mixture (1 ml, 1:1, v/v), centrifuging at 15,000 rpm for 5 min and removing supernatant from samples. After the last supernatant removal, samples were air dried (Sumiyoshi et al., 2013).

### Collecting Water Culture Media

After Al treatment, water culture media was frozen at −80°C and lyophilized (FDU-2200; Tokyo Rikakikai Co, Ltd) at 3.3 Pa for 8 days (Iwai et al., 2003).

### Determination of Uronic Acid

Uronic acid was determined by the method of Blumenkrantz and Gustav (1973). One milliliter of ion exchanged water was added into each sample, and 1 ml of iced concentrated sulfuric acid (0.025M borax) was mixed into 200 µl of each sample. After heating in 100°C water for 10 min and cooling in ice, 40 µl of carbazole solution (carbazole 125 mg/ethanol 100 ml) was mixed into each sample. Samples were heated in 100°C water for 15 min and cooled in ice to measure the absorbances at 530 nm (GENESIS 10S UV-VIS; Thermo Scientific).

## Results

### Distribution of Pectin in *star1* Mutant Roots After Al Treatment

**Figure 1:**
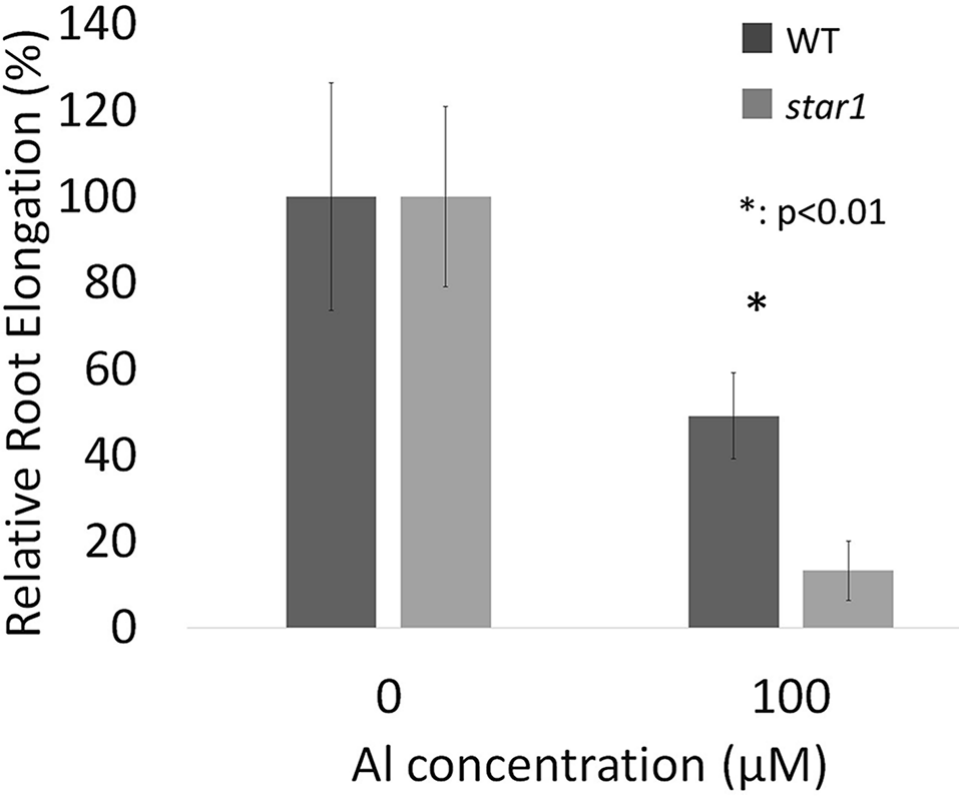
Relative root elongations (RREs) of WT (cv. Koshihikari) and *star1* seedlings during Al treatment (1.0 mM CaCl_2_, 0 or 100 µM AlCl_3_, pH 4.5). Root length of seedlings were measured before and after Al treatment and root elongations were calculated. Significant difference is shown between WT and *star1* under 100 µM Al treatment (Student’s *t*-test, *p* < 0.01). Data are means ± SD, n = 10.

**Figure 2:**
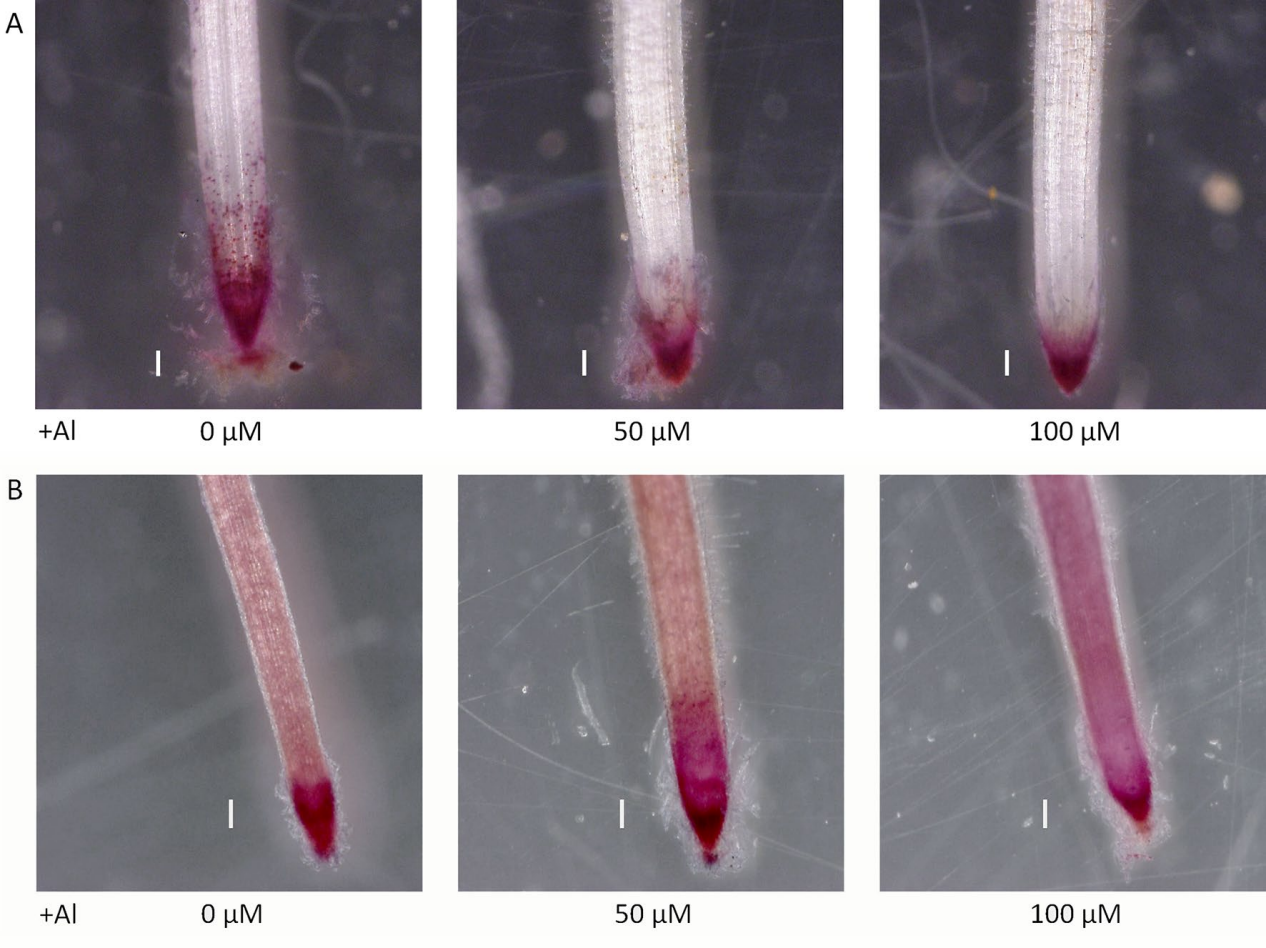
Demethylated pectin on ruthenium red staining without **(A)** and after **(B)** saponification (0.1 N NaOH, 1 min) in roots of WT (cv. Nipponbare) seedlings treated with Al (0, 50, or 100 µM). Roots were stained with 0.01% ruthenium red for 5 min. Bars = 0.1 mm.

**Figure 3:**
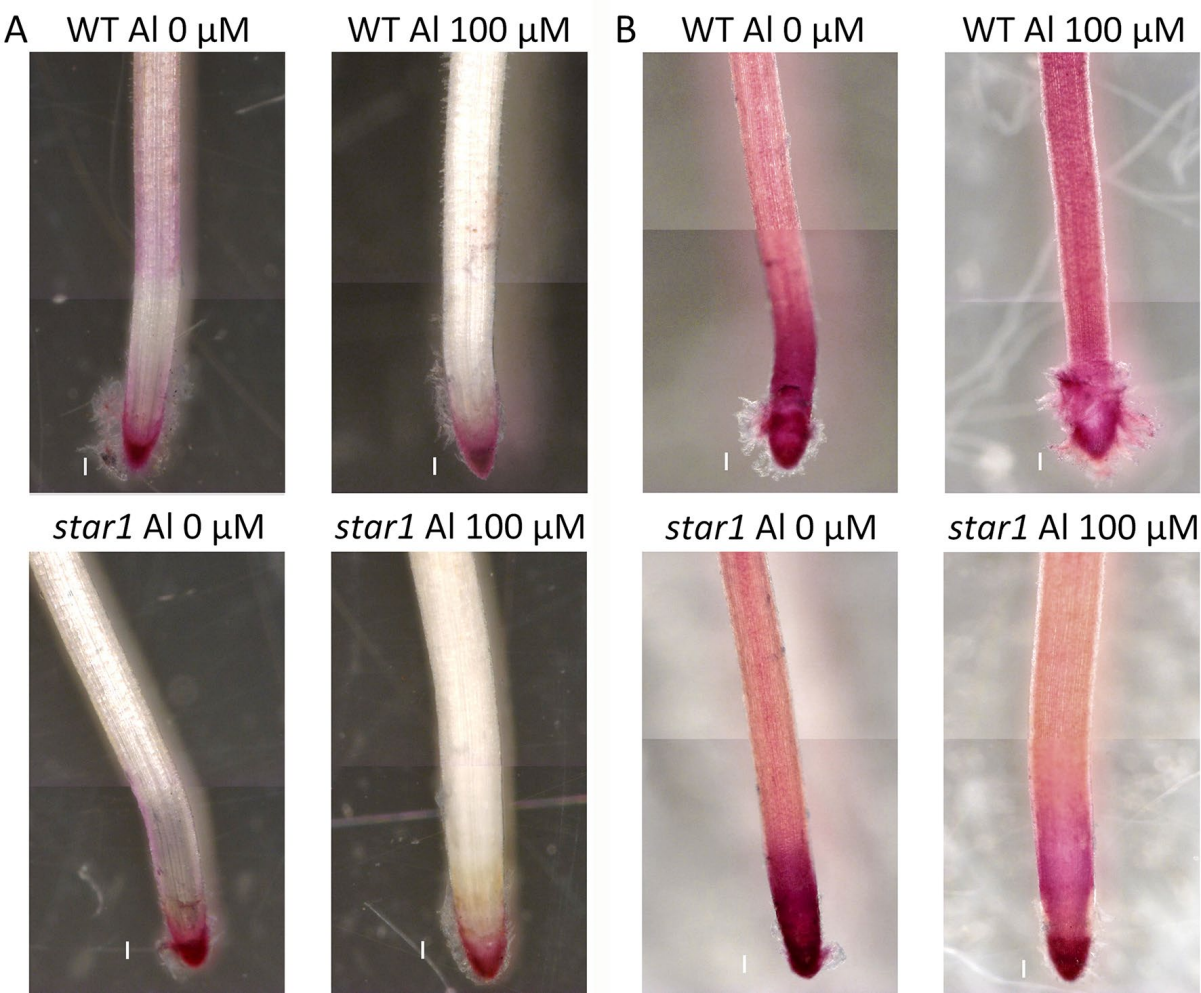
Demethylated pectin on ruthenium red staining without **(A)** and after **(B)** saponification (0.1 N NaOH, 1 min) in roots of WT (cv. Koshihikari) and *star1* seedlings treated with Al (0 or 100 µM). Roots were stained with 0.01% ruthenium red for 5 min. Bars = 0.1 mm.

Seedlings grown for 3 to 4 days with culture media containing 0.5 and 1.0 mM CaCl_2_ (pH 4.5) were treated for 1 day with culture media containing 0, 50, and 100 µM AlCl_3_. The elongation of roots during Al treatment was calculated using pre- and post-treatment root length measurements. Relative elongation of 100 µM Al-treated roots was reduced by about 70% in the *star1* mutant compared with the WT, and the root elongation of the *star1* mutant was significantly low (*t*-test, *p* < 0.01; **Figure 1**). After treatment, the control WT roots were stained with ruthenium red, indicating the presence of demethylesterified pectin, 0.2 mm from the root tips. Staining revealed the presence of demethylesterified pectin in the roots of the WT and *star1* mutant after Al treatment. RBCs of WT roots treated with 0-µM Al also showed staining. However, pectin staining distribution of WT RBCs and roots treated with 50- and 100-µM Al narrowed (**Figure 2A**). All Al treatments narrowed demethylesterified pectin staining distribution of STAR1 RBCs and roots (**Figure 3A**). Whole roots showed staining for saponified pectin (total pectin); in roots treated with high Al concentrations, an increase in staining was observed on the root side of the root cap (**Figure 2B**). In comparison with Al-treated WT (Koshihikari) roots, Al-treated roots STAR1 showed suppression of the increased staining in the root-side section beyond 1 mm from the tip (**Figure 3B**). A significant difference was not observed in the alteration in distribution of demethylesterified pectin (**Supplemental Figure 1A**) or all pectin (**Supplemental Figure 1B**) in roots by Al treatment between Nipponbare and Koshihikari.

### Distribution of Al in *star1* Mutant Roots After Al Treatment

**Figure 4:**
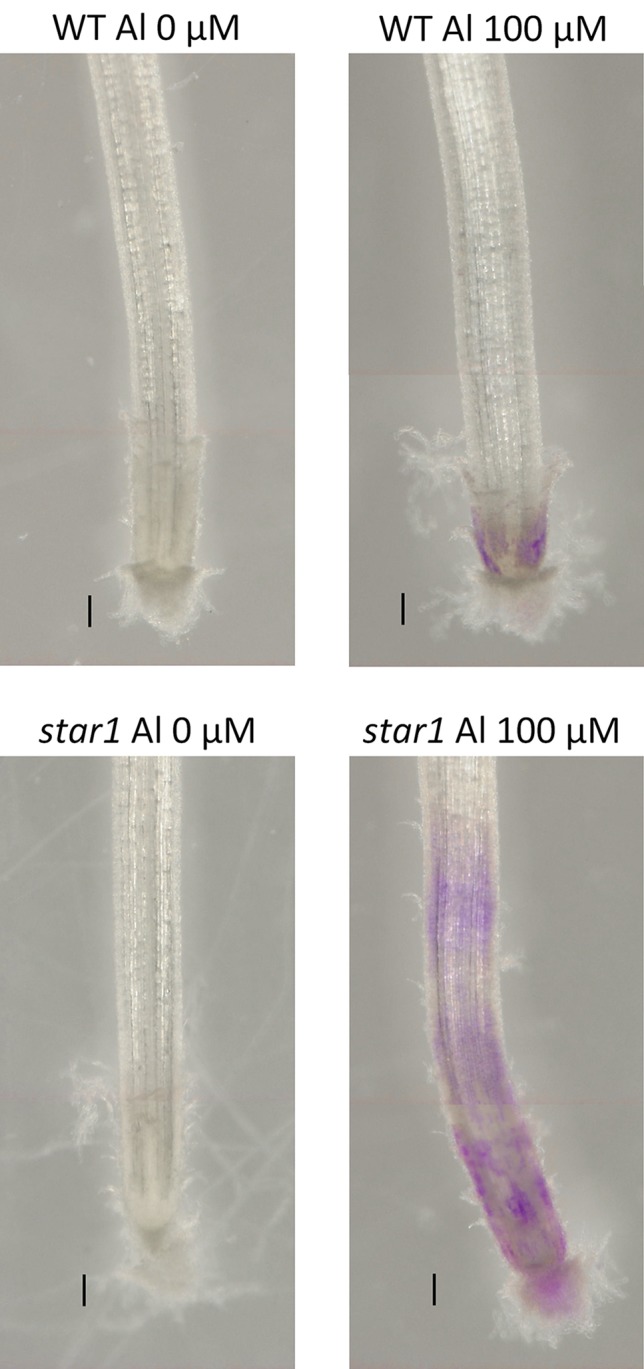
Al on eriochrome cyanine R staining in roots of WT (cv. Koshihikari) and *star1* seedlings treated with Al (0 or 100 µM for 24 h). Roots were stained with 0.1% eriochrome cyanine R for 20 min. Bars = 0.1 mm.

After Al treatment, the WT (Koshihikari) and STAR1 roots were stained with eriochrome cyanine R. Little staining was observed in the WT RBCs. In STAR1, strong staining by eriochrome cyanine R was observed in many regions of the root and RBCs (**Figure 4**). In particular, staining was stronger in RBCs than in other root regions.

### Quantification of Pectin in Cell Walls and Culture Media

**Figure 5:**
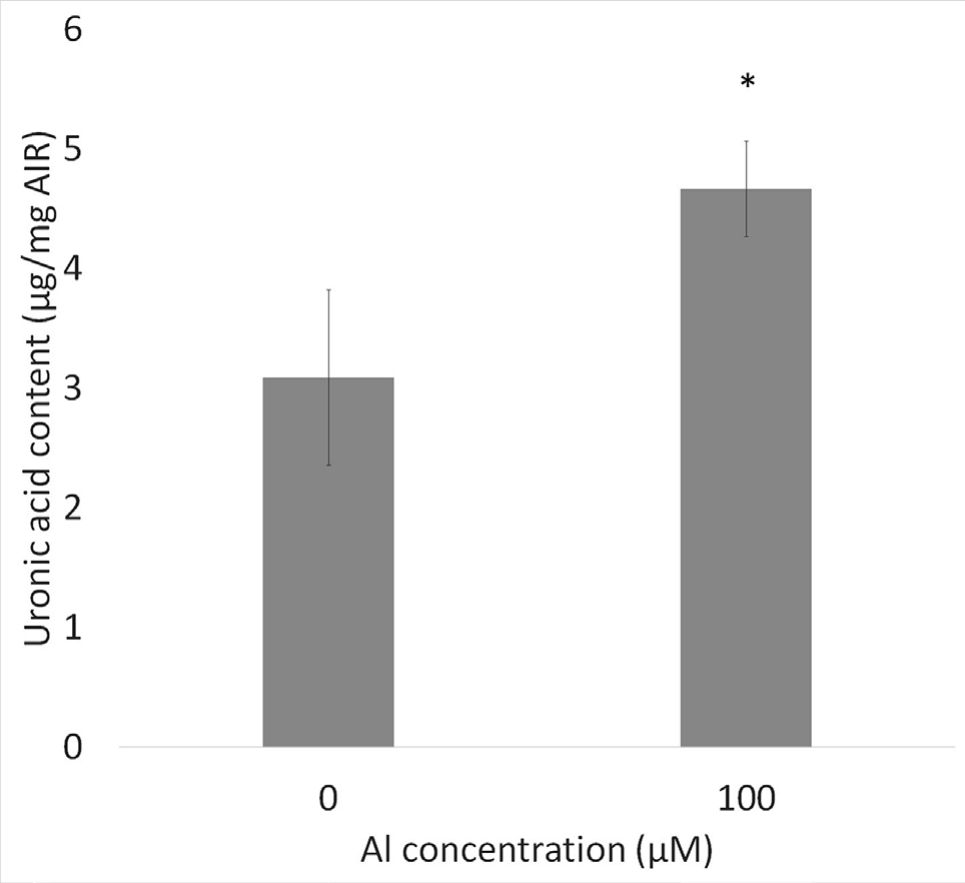
Uronic acid content in cell wall from root tips (0–1 mm) of WT (cv. Koshihikari) seedlings after Al treatment (1.0 mM CaCl_2_, 0 or 100 µM AlCl_3_, pH 4.5) for 24 h. Significant difference is shown between WT and *star1* under 100 µM Al treatment (Student’s *t*-test, *p* < 0.01). Data are means ± SD, n = 5.

The amounts of total pectin contained in the cell walls 1 mm from the root tips in control and Al-treated plants were quantified by the carbazole-sulfuric acid method. Pectin contents were increased by about 50% in Al-treated plants compared with the control, suggesting that Al treatment increased the pectin content in root ends (**Figure 5**).

**Figure 6:**
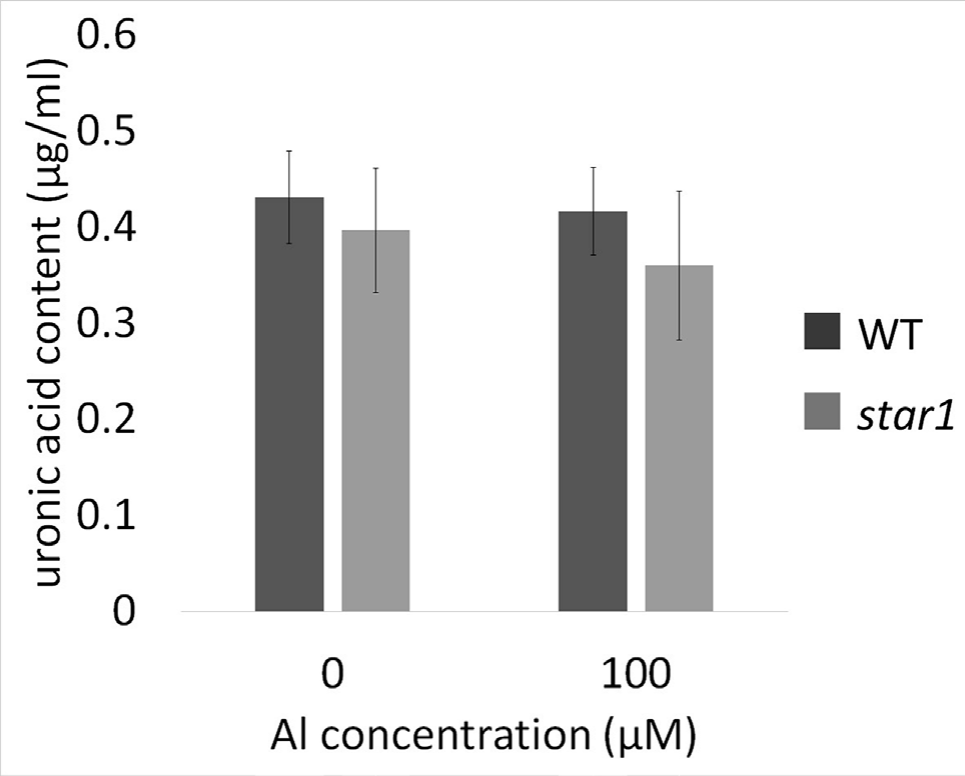
Uronic acid content in water culture media for WT (cv. Koshihikari) and *star1* collected after Al treatment (0 or 100 µM). Significant difference was not shown. Data are means ± SD, n = 6.

The pectin content of culture media did not differ significantly according to Al treatment or plant strain (**Figure 6**).

## Discussion

The root tip is the primary site of Al toxicity in higher plants (Ryan et al., 1993), and its encasing mucilaginous capsule has been implicated as a protective source of materials that prevent the uptake of Al into root meristems (Horst et al., 1982). To our knowledge, the physiological mechanism by which Al damages cells is not known (Kochian, 1995). The root cell wall has been suggested to be a site of Al toxicity and Al exclusion (Horst et al., 2010). Up to 90% of the Al absorbed by roots can be localized to the apoplast (Kochian, 1995). The primary site of Al^3+^ binding is probably the pectin matrix, which is composed largely of homopolymers of galacturonic acid (Mohnen, 2008; Horst et al., 2010). During plant development, cell wall pectin biosynthesis and assembly occur, and pectins secreted to the apoplast are highly esterified and later de-esterified by the activity of pectin methylesterase, inducing pectin–Ca crosslinking, which has an important role in the cell wall. Al^3+^ is known to bind far more strongly to pectin than to Ca^2+^, whose binding to the cell wall is required for proper cell wall functioning (Franco et al., 2002). Al treatment has been shown to increase the pectin content in the roots of pumpkin (Van et al., 1994), suggesting that pectin is involved in the Al stress response. In addition, the pectin distribution in response to Al and its possible impact on the Al resistance of RBCs had not been examined. In this study, an Al-sensitive mutant was used to test the hypothesis that RBCs and the pectin that they produce are involved in the detection and avoidance of Al toxicity. The Al concentration we used was higher than the exchangeable Al concentration in acid soil. However, some previous Al toxicity researches in rice employed around 100 µM Al^3+^ (Huang et al., 2009; Famoso et al., 2010; Cai et al., 2011; Maejima et al., 2014). Therefore, the range of Al concentration we used in this study is consistent with those.

The demethylesterified pectin distribution of roots, and especially of RBCs, narrowed with Al treatment in a dose-dependent manner (**Figure 2A**). Initially, we considered the hypothesis that the Al tolerance of rice could be increased by decreasing the demethylesterified pectin content. However, the type II cell walls of monocotyledonous plants, including rice, contain little pectin (Yokoyama and Nishitani, 2004); dicotyledonous plants with type I cell walls are pectin rich (Yang et al., 2011). Thus, proving that a high demethylesterified pectin content alone, which could increase the Al tolerance of rice, was difficult.

Therefore, we focused on the distribution and function, instead of the amount of demethylesterified pectin. Our results showed that RBC pectin distribution was related closely to Al concentrations in the culture media (**Figure 2A**). Therefore, active RBC release demethylesterified pectin in root tips, and subsequently, separated RBCs can absorb Al in culture media. This RBC behavior may block the accumulation of Al in root tips. However, the pectin content of culture media did not differ significantly according to the presence or absence of Al treatment in STAR1 or WT samples (**Figure 6**). Thus, demethylesterified pectin produced by RBCs and secreted in the culture medium does not contribute to the reduction of Al toxicity.

Hemicellulose is among the polysaccharide constituents of the cell wall. This polysaccharide is fractionated from the cell wall using potassium hydroxide after the fractionation of pectin (Yang et al., 2011; Zhu et al., 2017a; Zhu et al., 2017b). Hemicellulose interacts with cellulose fiber by hydrogen bonding and is covalently bonded to hemicellulose on neighboring cellulose fibers, forming a chain of pectic polymers (Scheller and Ulvskov, 2010). Hemicellulose polysaccharides are classified as xyloglucan, xylan, β-(1–3), (1–4)-glucan, callose, and arabinogalactan, and they play an important role in cell elongation *via* cell wall loosening (Scheller and Ulvskov, 2010). The cell walls of monocotyledonous plants, including rice, contain 30% to 70% hemicellulose in their primary cell walls (Scheller and Ulvskov, 2010). As hemicellulose has been found to adsorb Al preferentially to pectin (Yang et al., 2011; Zhu et al., 2017a; Zhu et al., 2017b), we compared the distributions of demethylesterified pectin, total pectin, and adsorbed Al. On eriochrome cyanine R staining, the region that adsorbs Al appeared not to overlap with the region that contained demethylesterified pectin or total pectin. In WT plants containing high levels of demethylesterified pectin, Al-treated roots showed almost no accumulation of Al (**Figure 4**). On the other hand, Al accumulation was found in a wide range of root caps and RBCs in the *star1* mutant (**Figure 4**). Although the distribution of demethylesterified pectin in roots was detected in the WT (**Figures 2A** and **3A**), these roots showed little Al accumulation (**Figure 4**). These results suggest that demethylesterified pectin in WT RBCs is crosslinked mainly with Ca and linked slightly to Al^3+^. Al^3+^ may stick to Ca-bonded pectin, which acts as a cation exchange resin because Ca bonding of pectin is irreversible and Al^3+^ binding to cell-wall pectin through replacement of Ca^2+^ is impossible. In addition, alkali-soluble pectin contained in RBCs appears to adsorb Al in the early stage of growth inhibition in pea (Yang et al., 2016). Pectin responsible for cell wall adhesion between cells is actively demethylesterified by pectin methylesterase (PME) and degraded by polygalacturonase (PG), which is thought to cause RBCs to fall out of the root cap (Stephenson and Hawes, 1994; Wen et al., 1999). RBCs, including those with Al bound to Ca-bonded pectin, were separated and released to the culture medium. On the other hand, the accumulation of Al in hemicellulose has been suggested to increase in the AtSTAR1 function-deficient mutant of *A. thaliana* (Xu et al., 2018). Our results suggest that RBCs in the *star1* mutant have less pectin. Therefore, the hemicellulose, rather than pectin, in the cell walls of STAR1 accumulates Al. Our results are consistent with previous reports that Al-sensitive mutants and cultivars accumulate more Al than do controls (Ma et al., 2002; Ma et al., 2005) (**Figure 4**). Rice PME expression increases in Al-treated roots; Al accumulation in roots increases in PME-overexpressing rice, and Al tolerance decreases (Yang et al., 2013). Pectin was actively demethylesterified by overexpression of PME, became susceptible to decomposition by PG, and its root content was reduced while RBCs elimination was promoted, increasing Al resistance through the pectin in RBCs. The function of this mechanism is considered to decrease with increasing Al accumulation (Yang et al., 2013). Our results suggest that Al accumulation in rice roots is reduced by the distribution of pectin in root tips, and that pectin in the root cell walls contributes to the acquisition of Al tolerance in rice by regulating its quantity and distribution. These pectin-related barriers to Al reduce Al toxicity. The release of Al-binding mucilage by RBCs could play a role in protecting root tips from Al-induced cellular damage.

## Data Availability Statement

The datasets generated for this study can be found in University of Tsukuba Repository, https://tsukuba.repo.nii.ac.jp.

## Author Contributions

TN, AN, JF, and HI conceived the experiment(s), analyzed the result(s), and wrote the paper. JF and HI conducted the experiment(s). All authors reviewed the manuscript.

## Conflict of Interest

The authors declare that the research was conducted in the absence of any commercial or financial relationships that could be construed as a potential conflict of interest.

## Supplementary Material

Figure S1 Demethylated pectin on ruthenium red staining without **(A)** and after **(B)** saponification (0.1 N NaOH 1 min) in roots of WT (cv. Koshihikari) seedlings treated with Al (0, 100 µM). Roots were stained with 0.01% ruthenium red for 5 min. Bars = 0.1 mm.

## References

[B1] Barcelo J. Poschenrieder C. (2002). Fast root growth responses, root exudates, and internal detoxification as clues to the mechanisms of aluminium toxicity and resistance: a review. Environ. Exp. Bot. 48 1, 75–92. 10.1016/S0098-8472(02)00013-8

[B2] Blumenkrantz N. Gustav A.H. (1973) New method for quantitative determination of uronic acids. Analytical Biochemistry 54 2, 484–489. 10.1016/0003-2697(73)90377-1 4269305

[B3] Cai M. Z. Zhang S. N. Xing C. H. Wang F. M. Wang N. Zhu L. (2011). Developmental characteristics and aluminum resistance of root border cells in rice seedlings. Plant Sci. 180 5, 702–708. 10.1016/j.plantsci.2011.01.017 21421421

[B4] Chang Y.-C. Yamamoto Y. Matsumoto H. (1999). Accumulation of aluminium in the cell wall pectin in cultured tobacco (*Nicotiana tabacum* L.) cells treated with a combination of aluminium and iron. Plant Cell Environ. 22 8, 1009–1017. 10.1046/j.1365-3040.1999.00467.x

[B5] Curlango-Rivera G. Gunawardena U. Wen F. Zhao X. Xion Z. Hawes M. C. (2016). Roots: contribution to the rhizosphere. eLS. 10.1002/9780470015902.a0002335.pub3

[B6] Delhaize E. Ma J. F. Ryan P. R. (2012). Transcriptional regulation of aluminium tolerance genes. Trends Plant Sci. 17 6, 341–348. 10.1016/j.tplants.2012.02.008 22459757

[B7] Duan J. Gregory J. (2003). Coagulation by hydrolysing metal salts. Adv. Colloid Interface Sci. 100, 475–502. 10.1016/S0001-8686(02)00067-2

[B8] Famoso A. N. Randy T. C. Jon E. S. Eric C. Susan R. M. Leon V. K. (2010). Development of a novel aluminum tolerance phenotyping platform used for comparisons of cereal Al tolerance and investigations into rice Al tolerance mechanisms. Plant Physiol. 153 4, 1678–1691. 10.1104/pp.110.156794 20538888 PMC2923895

[B9] Foy C. D. (1988). Plant adaptation to acid, aluminum-toxic soils. Commun. Soil Sci. Plant Anal. 19 7-12, 959–987. 10.1080/00103628809367988

[B10] Franco C. R. Chagas A. P. Jorge R. A. (2002). Ion-exchange equilibria with aluminum pectinates. Colloids Surf. A Physicochem. Eng. Asp. 204 1-3, 183–192. 10.1016/S0927-7757(01)01134-7

[B11] Furukawa J. Yamaji N. Wang H. Mitani N. Murata Y. Sato K. (2007). An aluminum-activated citrate transporter in barley. Plant Cell Physiol. 8, 1081–1091. 10.1093/pcp/pcm091 17634181

[B12] Gunawardena U. Hawes M. C. (2002). Tissue specific localization of root infection by fungal pathogens: role of root border cells. Mol. Plant Microbe Interact. 15 11, 1128–1136. 10.1094/MPMI.2002.15.11.1128 12423018

[B13] Hawes M. C. Lin H. J. (1990). Correlation of pectolytic enzyme activity with the programmed release of cells from root caps of pea (*Pisum sativum*). Plant Physiol. 94 4, 1855–1859. 10.1104/pp.94.4.1855 16667927 PMC1077464

[B14] Hawes M. C. Gunawardena U. Miyasaka S. Zhao X. (2000). The role of root border cells in plant defense. Trends Plant Sci. 5 3, 128–133. 10.1016/S1360-1385(00)01556-9 10707079

[B15] Hawes M. C. Lain J. M. Ramirez-Andreotta M. Curlango-Rivera G. Flores-Lara Y. Brigham L. A. (2016). Extracellular trapping of soil contaminants by root border cells: new insights into plant defense. Agronomy 6 1 5, 1–9. 10.3390/agronomy6010005

[B16] Hoekenga O. A. Maron L. G. Piñerous M. A. Cançado G. M. A. Shaff J. Kobayashi Y. (2006). *AtALMT1*, which encodes a malate transporter, is identified as one of several genes critical for aluminum tolerance in *Arabidopsis* . Proc. Nat. Acad. Sci. 103 25, 9738–9743. 10.1073/pnas.0602868103 16740662 PMC1480476

[B17] Horst W. J. Wagner A. Marschner H. (1982). Mucilage protects root meristems from aluminium injury. Zeitschrift für Pflanzenphysiologie 105 5, 435–444. 10.1016/S0044-328X(82)80041-X

[B18] Horst W. J. Wang Y. Eticha D. (2010). The role of the root apoplast in aluminium-induced inhibition of root elongation and in aluminium resistance of plants: a review. Ann. Bot. 106 1, 185–197. 10.1093/aob/mcq053 20237112 PMC2889789

[B19] Huang C. F. Yamaji N. Mitani N. Yano M. Nagamura Y. Ma J. F. (2009). A bacterial-type ABC transporter is involved in aluminum tolerance in rice. Plant Cell 21 2, 655–667. 10.1105/tpc.108.064543 19244140 PMC2660611

[B20] Iwai H. Kikuchi A. Kobayashi T. Kamada H. Satoh S. (1999). High levels of non-methylesterified pectins and low levels of peripherally located pectins in loosely attached non-embryogenic callus of carrot. Plant Cell Rep. 18, 561–566. 10.1007/s002990050622

[B21] Iwai H. Usui M. Hoshino H. Kamada H. Matsunaga T. Kakegawa K. (2003). Analysis of sugars in squash xylem sap. Plant Cell Physiol. 44 6, 582–587. 10.1093/pcp/pcg075 12826623

[B22] Jones L. H. Thurman D. A. (1957). The determination of aluminium in soil, ash and plant materials using Eriochrome Cyanine R.A. Plant Soil 9 2, 131–142. 10.1007/BF01398921

[B23] Jones D. L. Blancaflor E. B. Kochian L. V. Gilroy S. (2006). Spatial coordination of aluminium uptake, production of reactive oxygen species, callose production and wall rigidification in maize roots. Plant Cell Environ. 29 7, 1309–1318. 10.1111/j.1365-3040.2006.01509.x 17080952

[B24] Kochian L. V. (1995). Cellular mechanisms of aluminum toxicity and resistance in plants. Annu. Rev. Plant Biol. 46 1, 237–260. 10.1146/annurev.pp.46.060195.001321

[B25] Kochian L. V. Piñeros M. A. Hoekenga O. A. (2005). “The physiology, genetics and molecular biology of plant aluminum resistance and toxicity.” in Root physiology: from gene to function, Dordrecht: Springer, 175–195. 10.1007/s11104-005-0964-x

[B26] Kopittke P. M. McKenna B. A. Karunakaran C. Dynes J. J. Arthur Z. Gianoncelli A. (2017). Aluminum complexation with malate within the root apoplast differs between aluminum resistant and sensitive wheat lines. Front. Plant Sci. 8, 1377. 10.3389/fpls.2017.01377 28824696 PMC5541250

[B27] Li X. F. Ma J. F. Matsumoto H. (2000). Pattern of aluminum-induced secretion of organic acids differs between rye and wheat. Plant Physiol. 123 4, 1537–1544. 10.1104/pp.123.4.1537 10938369 PMC59110

[B28] Ma J. F. Nagao S. Huang C. F. Nishimura M. (2005). Isolation and characterization of a rice mutant hypersensitive to Al. Plant Cell Physiol. 46 7, 1054–1061. 10.1093/pcp/pci116 15857838

[B29] Ma J. F. Shen R. Zhao Z. Wissuwa M. Takeuchi Y. Ebitani T. (2002). Response of rice to Al stress and identification of quantitative trait loci for Al tolerance. Plant Cell Physiol. 43 6, 652–659. 10.1093/pcp/pcf081 12091719

[B30] Ma J. F. Ryan P. R. Delhaize E. (2001). Aluminium tolerance in plants and the complexing role of organic acids. Trends Plant Sci. 6 6, 273–278. 10.1016/S1360-1385(01)01961-6 11378470

[B31] Ma J. F. (2000). Role of organic acids in detoxification of aluminum in higher plants. Plant Cell Physiol. 41 4, 383–390. 10.1093/pcp/41.4.383 10845450

[B32] Maejima E. Watanabe T. Osaki M. Wagatsuma T. (2014). Phosphorus deficiency enhances aluminum tolerance of rice (*Oryza sativa*) by changing the physicochemical characteristics of root plasma membranes and cell walls. J. Plant Physiol. 171 2, 9–15. 10.1016/j.jplph.2013.09.012 24331414

[B33] Mohnen D. (2008). Pectin structure and biosynthesis. Curr. Opin. Plant Biol. 11 3, 266–277. 10.1016/j.pbi.2008.03.006 18486536

[B34] Parra-Almuna L. Diaz-Cortez A. Ferrol N. Mora M. L. (2018). Aluminium toxicity and phosphate deficiency activates antioxidant systems and up-regulates expression of phosphate transporters gene in ryegrass (*Lolium perenne* L.) plants. Plant Physiol. Biochem. 130, 445–454. 10.1016/j.plaphy.2018.07.031 30077920

[B35] Rengel Z. (2004). Aluminium cycling in the soil-plant-animal-human continuum. Biometals 17 6, 669–689. 10.1007/s10534-004-1201-4 15689110

[B36] Ryan P. R. Delhaize E. Jones D. L. (2001). Function and mechanism of organic anion exudation from plant roots. Annu. Rev. Plant Biol. 52 1, 527–560. 10.1146/annurev.arplant.52.1.527 11337408

[B37] Ryan P. R. Ditomaso J. M. Kochian L. V. (1993). Aluminium Toxicity in Roots: An Investigation of Spatial Sensitivity and the Role of the Root Cap. J. Exp. Bot. 44 2, 437–446. 10.1093/jxb/44.2.437

[B38] Scheller H. V. Ulvskov P. (2010). Hemicelluloses. Annu. Rev. Plant Biol. 61, 263–289. 10.1146/annurev-arplant-042809-112315 20192742

[B39] Searcy K. B. Mulcahy D. L. (1990). Comparison of the response to aluminum toxicity in gametophyte and sporophyte of four tomato (*Lycopersicon esculentum* Mill.) cultivars. Theor. Appl. Genet. 80 3, 289–295. 10.1007/BF00210062 24220959

[B40] Shaff J.E. Schultz B.A. Craft E.J. Clark R.T. Kochian L.V. (2010). GEOCHEM-EZ: a chemical speciation program with greater power and flexibility Plant Soil 330 1, 207–214. 10.1007/s11104-009-0193-9.

[B41] Stephenson M. B. Hawes M. C. (1994). Correlation of pectin methylesterase activity in root caps of pea with root border cell separation. Plant Physiol. 106 2, 739–745. 10.1104/pp.106.2.739 12232366 PMC159582

[B42] Sumiyoshi M. Nakamura A. Nakamura H. Hakata M. Ichikawa H. Hirochika H. (2013). Increase in cellulose accumulation and improvement of saccharification by overexpression of arabinofuranosidase in rice. PLoS One 8 11, e78269. 10.1371/journal.pone.0078269 24223786 PMC3817243

[B43] Tsutsui T. Yamaji N. Huang C. F. Motoyama R. Nagamura Y. Ma J. F. (2012). Comparative genome-wide transcriptional analysis of Al-responsive genes reveals novel Al tolerance mechanisms in rice. PLoS One 7 10, e48197. 10.1371/journal.pone.0048197 23110212 PMC3482186

[B44] Van H. L. Kurashiki S. Sakurai N. (1994). Aluminum-induced rapid root inhibition and changes in cell-wall components of squash seedlings. Plant Physiol. 106 3, 971–976. 10.1104/pp.106.3.971 12232377 PMC159620

[B45] Von Uexküll H. R. Mutert E. (1995). Global extent, development and economic impact of acid soils. Plant Soil 171 1, 1–15. 10.1007/BF00009558

[B46] Wang H. Chen R. F. Iwashita T. Shen R. F. Ma J. F. (2015). Physiological characterization of aluminum tolerance and accumulation in tartary and wild buckwheat. New Phytol. 205 1, 273–279. 10.1111/nph.13011 25195800

[B47] Watson B. S. Bedair M. F. Urbanczyk-Wochniak E. Huhman D. V. Yang D. S. Allen S. N. (2015). Integrated metabolomics and transcriptomics reveal enhanced specialized metabolism in *Medicago truncatula* root border cells. Plant Physiol. 167 4, 1699–1716. 10.1104/pp.114.253054 25667316 PMC4378151

[B48] Wen F. Zyu Y. Hawes M. C. (1999). Effect of pectin methylesterase gene expression on pea root development. Plant Cell 11 6, 1129–1140. 10.1105/tpc.11.6.1129 10368183 PMC144245

[B49] Xing C. H. Zhu M. H. Cai M. Z. Liu P. Xu G. D. Wu S. H. (2008). Developmental characteristics and response to iron toxicity of root border cells in rice seedlings. J. Zhejiang Univ. Sci. B 9 3, 261–264. 10.1631/jzus.B0710627 18357629 PMC2266877

[B50] Xu J. M. Lou H. Q. Jin J. F. Chen W. W. Wan J. X. Fan W. (2018). A half-type ABC transporter FeSTAR1 regulates Al resistance possibly *via* UDP-glucose-based hemicellulose metabolism and Al binding. Plant Soil 432 1–2, 303–314. 10.1007/s11104-018-3805-4

[B51] Yang J. L. Zhu X. F. Peng Y. X. Zheng C. Li G. X. Liu Y. (2011). Cell wall hemicellulose contributes significantly to Al adsorption and root growth in Arabidopsis. Plant Physiol. 155 4, 1885–1892. 10.1104/pp.111.172221 21285327 PMC3091086

[B52] Yang J. Qu M. Fang J. Shen R. F. Feng Y. M. Liu J. Y. (2016). Alkali-soluble pectin is the primary target of aluminum immobilization in root border cells of pea (*Pisum sativum*). Front. Plant Sci. 7, 1297. 10.3389/fpls.2016.01297 27679639 PMC5020075

[B53] Yang X. Y. Zeng Z. H. Yan J. Y. Fan W. Bian H. W. Zhu M. Y. (2013). Association of specific pectin methylesterases with Al-induced root elongation inhibition in rice. Physiol. Plant. 148 4, 502–511. 10.1111/ppl.12005 23136980

[B54] Yokosho K. Naoki Y. Miho F. K. Jian F. M. (2016). Functional analysis of a MATE gene *OsFRDL2* revealed its involvement in Al-induced secretion of citrate, but a lower contribution to Al tolerance in rice. Plant Cell Physiol. 57 5, 976–985. 10.1093/pcp/pcw026 26872836

[B55] Yokoyama R. Nishitani K. (2004). Genomic basis for cell-wall diversity in plants. A comparative approach to gene families in rice and Arabidopsis. Plant Cell Physiol. 45 9, 1111–1121. 10.1093/pcp/pch151 15509833

[B56] Yoshida S. Forno D.A. Cock J.H. Gomez K.A. (1972) Laboratory Manual for Physiological Studies of Rice, 2nd Ed., The International Rice Research Institute, Los Baños, Philippines 53–57.

[B57] Yu M. Shen R. Liu J. Chen R. Xu M. Yang Y. (2009). The role of root border cells in aluminum resistance of pea (*Pisum sativum*) grown in mist culture. J. Plant Nutrit. Soil Sci. 172 4, 528–534. 10.1002/jpln.200800039

[B58] Zhu X. F. Zhao X. S. Wang B. Wu Q. Shen R. F. (2017). Elevated carbon dioxide alleviates aluminum toxicity by decreasing cell wall hemicellulose in rice (*Oryza sativa*). Front. Physiol. 8, 512. 10.3389/fphys.2017.00512 28769823 PMC5513963

[B59] Zhu X. F. Wan J. X. Wu Q. Zhao X. S. Zheng S. J. Shen R. F. (2017b). *PARVUS* affects aluminium sensitivity by modulating the structure of glucuronoxylan in *Arabidopsis thaliana* . Plant Cell Environ. 40 9, 1916–1925. 10.1111/pce.12999 28622705

