# Supplementary figures and images for: Changes in the Distribution of Pectin in Root Border Cells Under Aluminum Stress
Source: Front Plant Sci. 2019 Oct 2;10:1216. doi: 10.3389/fpls.2019.01216 (PMC6783878; doi:10.3389/fpls.2019.01216)

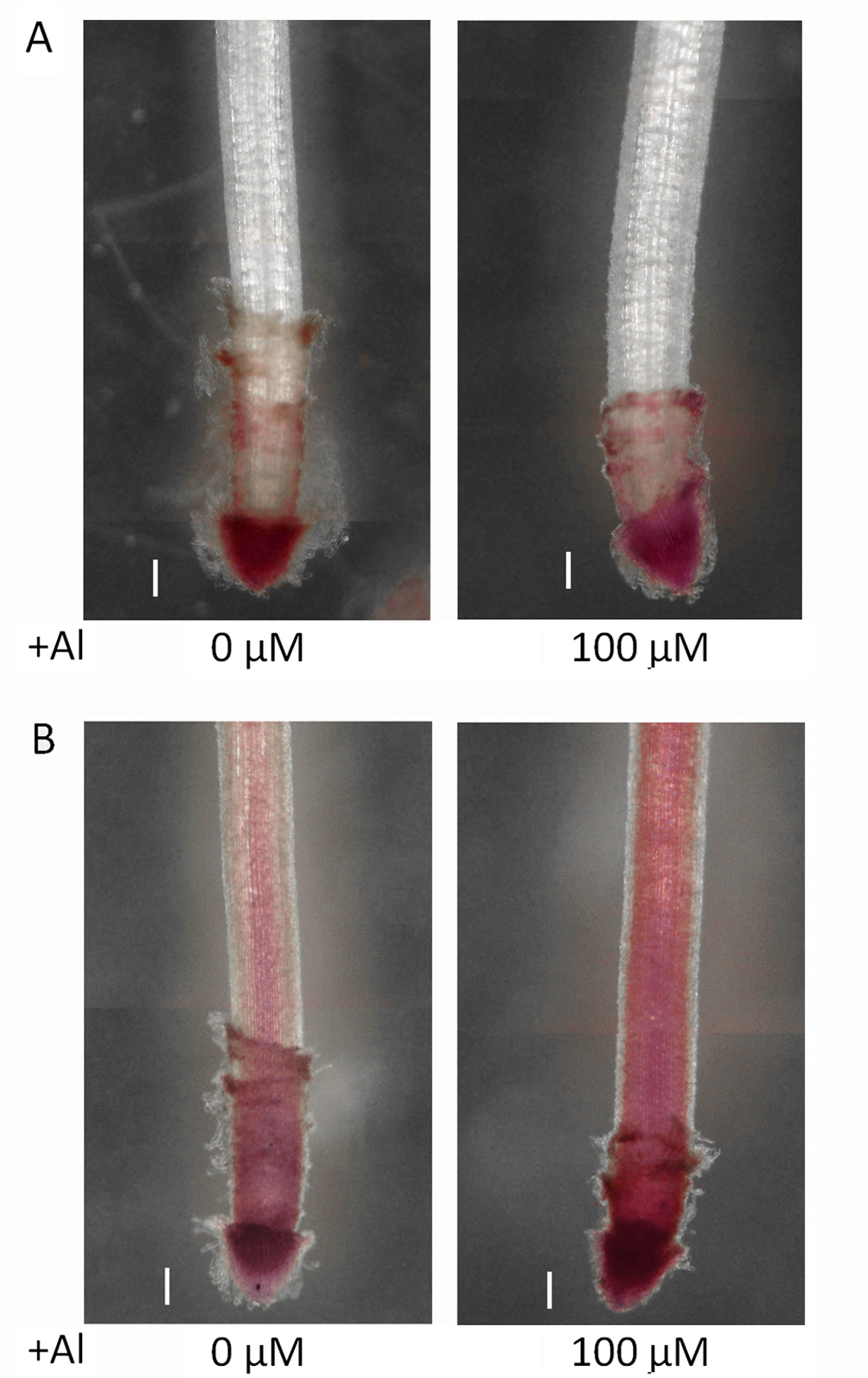

Supplement: Figure S1 — Demethylated pectin on ruthenium red staining without (A) and after (B) saponification (0.1 N NaOH 1 min) in roots of WT (cv. Koshihikari) seedlings treated with Al (0, 100 µM). Roots were stained with 0.01% ruthenium red for 5 min. Bars = 0.1 mm. [file Image_1.tif]
